# Causal relationship between multiple sclerosis and primary Sjögren’s syndrome: a two-sample mendelian randomization study

**DOI:** 10.1007/s11011-024-01379-8

**Published:** 2024-07-17

**Authors:** Jie Shen, Qiao Ye, Fang Luo, Tianhang Yu, Jinli Miao, Wenmin Wang, Hui Yuan

**Affiliations:** 1 Department of Rheumatology and Immunology, The Second Affiliated Hospital of Jiaxing University, Jiaxing, 314000 Zhejiang China; 2 The Yangtze River Delta Biological Medicine Research and Development Center of Zhejiang Province, Yangtze Delta Region Institution of Tsinghua University, Hangzhou, 314006 Zhejiang China; 3 Department of Logistics Support, Jiaxing Hospital of Traditional Chinese Medicine, Jiaxing, 314000 Zhejiang China

**Keywords:** Primary Sjögren's syndrome, Multiple sclerosis, Mendelian randomization, Genetic analysis, Autoimmune diseases

## Abstract

This study aims to investigate the causal relationship between primary Sjögren’s syndrome (SS) and multiple sclerosis (MS) using a two-sample Mendelian randomization (MR) analysis to provide insights into their common mechanisms and implications for therapeutic strategies. We utilized data from Genome-Wide Association Studies (GWAS) for primary SS (1,290 cases and 213,145 controls) and MS (4,888 cases and 10,395 controls), restricted to European ancestry. Instrumental variables (IVs) were selected based on genetic variants associated with primary SS. The primary MR method was Inverse Variance Weighted (IVW), supplemented by MR Egger, Weighted Median, Simple Mode, and Weighted Mode algorithms to assess the bidirectional causal relationships between MS and primary SS. Sensitivity analyses, including MR-PRESSO and leave-one-out analysis, were conducted to ensure the robustness of our findings. After excluding SNPs with pleiotropic effects, 42 and 5 SNPs were identified as robust IVs for primary SS and MS, respectively. Our analysis revealed a significant protective effect of MS on primary SS, with IVW showing an OR of 0.896 (95% CI: 0.841–0.954, *P* = 0.001). No significant heterogeneity or horizontal pleiotropy was detected, supporting the reliability of the results. Our findings suggest a potential protective effect of MS against primary SS, indicating a negative causal association between these two autoimmune diseases. This adds valuable genetic evidence to the understanding of the complex interplay between primary SS and MS, offering new avenues for research and therapeutic interventions.

## Introduction

Primary Sjögren’s syndrome (SS) is an autoimmune disorder characterized by the dysfunction of exocrine glands, leading to the hallmark symptoms of dry mouth and eyes. Epidemiological studies have established primary SS as a condition with variable global incidence and prevalence, highlighting its impact on both society and healthcare systems. While these studies have provided insights into the disease’s demographics (Qin et al. 2015), revealing a pooled incidence rate of 6.92 per 100,000 person-years and a prevalence rate of 60.82 per 100,000, with a significant female predominance. This distribution highlights the need for targeted research and intervention strategies to address the healthcare burden posed by primary SS.

Multiple sclerosis (MS) is another chronic, immune-mediated disorder, primarily affecting the central nervous system. It leads to neural demyelination, resulting in a wide range of neurological symptoms, including motor, sensory, and cognitive impairments. The global prevalence of MS has been rising, with an estimated 2.8 million people living with the condition worldwide, translating to about 35.9 per 100,000 population. This increase in prevalence has been observed across every world region since 2013, indicating a significant global disease burden. Moreover, the incidence rate across 75 reporting countries is approximately 2.1 per 100,000 person-years, with the mean age of diagnosis around 32 years, and the prevalence in females is double that of male(Walton et al. 2020). Like primary SS, MS presents a considerable disease burden worldwide, with varying incidence and prevalence that reflect its significant impact on individuals and healthcare infrastructures. The global prevalence of MS underscores the urgency of advancing our understanding and treatment of this debilitating condition.

Emerging evidence suggests a potential link between primary SS and MS, with several studies indicating an overlap in immunological features and genetic predispositions (Sanchez-Cerrillo et al. 2023). Notably, the convergence of these disorders is marked by their shared immunopathological underpinnings, with evidence pointing towards a synergistic exacerbation of autoimmune responses. This shared pathology invites a deeper inquiry into the molecular and cellular mechanisms bridging primary SS and MS, suggesting a complex interplay that might illuminate novel therapeutic targets (Andre and Bockle 2022; Correale et al. 2017; Hong et al. 2022). The intersection of primary SS and MS, characterized by distinct yet overlapping disease phenotypes, underscores the imperative for advanced diagnostic criteria and tailored treatment modalities, leveraging the shared immunoregulatory dysfunctions to foster innovative approaches in managing these conditions.

In this context, Mendelian randomization (MR) offers a method to investigate the potential causal relationship between these diseases. By using genetic variation as an instrumental variable, MR can differentiate direct associations between two diseases from those resulting from shared risk factors (Sekula et al. 2016). This approach relies on the premise that individuals inherit genetic variants influencing the risk of specific conditions (e.g., SS) through random assignment at conception, akin to the randomization in controlled trials. This mechanism offers a novel perspective on studying causal links between primary SS and MS. By leveraging large-scale genome-wide association studies (GWAS) data for both conditions, our study aims to employ MR to elucidate the potential causal pathways linking primary SS and MS (Burgess and Thompson 2017). This investigation is expected to provide novel insights into their common mechanisms and inform the development of more effective therapeutic strategies, ultimately improving outcomes for patients suffering from these conditions.

## Methods

### Data sources

**Fig. 1:**
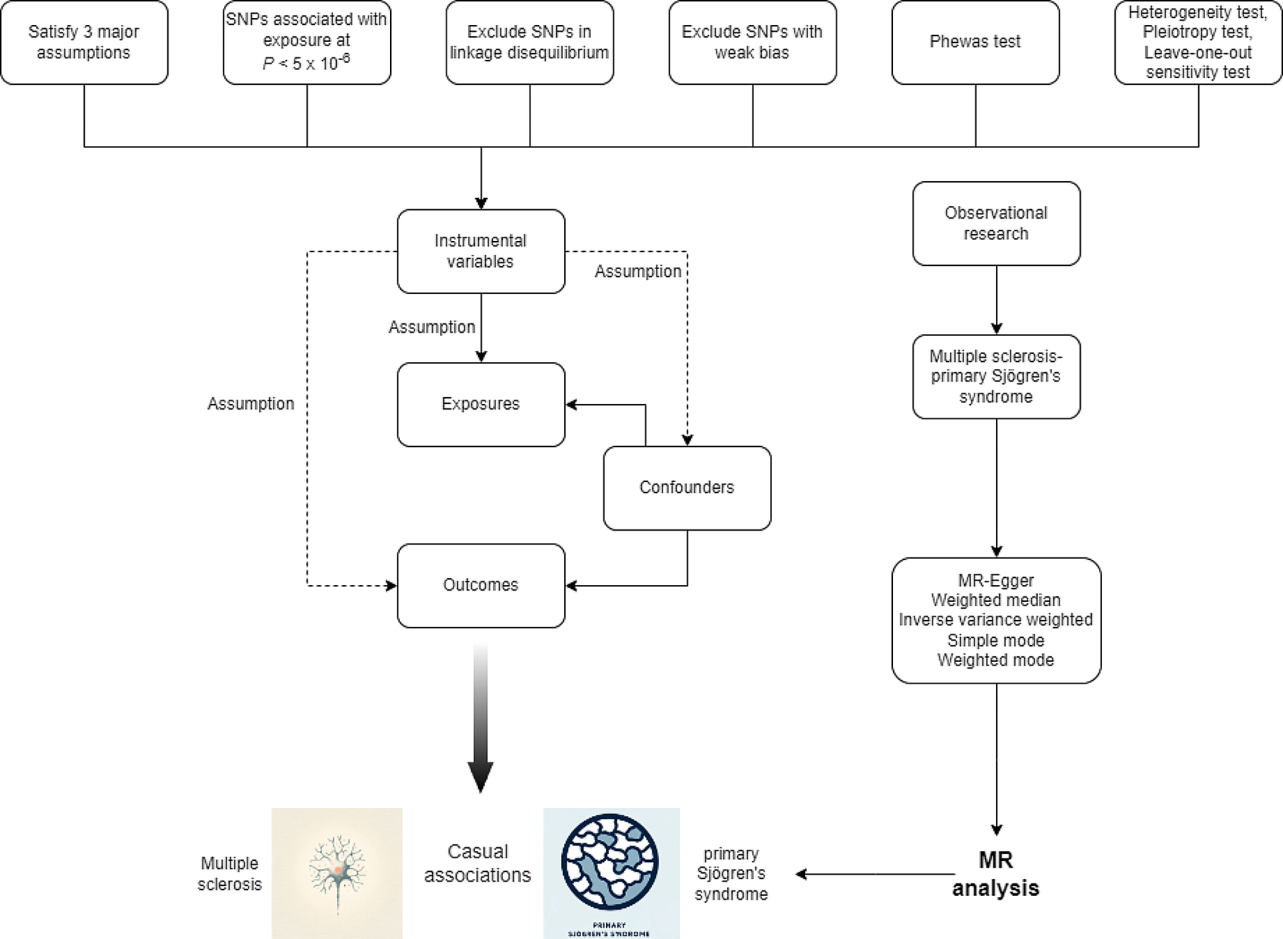
The flowchart of MR analysis. This flowchart presents the MR analysis process used to investigate the causal relationship between MS and primary SS. The process begins by satisfying three major assumptions for MR studies. SNPs are selected based on their association with the exposure (*P* < 5 × 10^−8^) and are further refined by excluding SNPs in linkage disequilibrium and those with weak bias. Instrumental variables are identified for the exposures of interest, accounting for potential confounders. Observational research data is incorporated to support the MR findings. The outcomes of the MR analysis are then evaluated using several methods: MR-Egger, weighted median, inverse variance weighted, simple mode, and weighted mode, to ensure robustness and minimize bias

In this study, we conducted an extensive search of databases generated from Genome-Wide Association Studies (GWAS), including GWAS Catalog, IEU openGWAS, and Neale Lab, from which we extracted eligible datasets. To mitigate the bias caused by race-related confounding factors, our research population was confined to European ancestry. Specifically, the data for Multiple Sclerosis (MS, 4,888 cases and 10,395 controls) were sourced from the European Bioinformatics Institute (EBI) (Sollis et al. 2023), and the data for primary SS (1,290 cases and 213,145 controls) came from the FinnGen Biobank (Andlauer et al. 2016). The case definitions for MS and primary SS adhered to the International Classification of Diseases 10 (ICD-10). This study was reported following the STROBE-MR guidelines (Skrivankova et al. 2021), and all the utilized data have been published in the public domain, obviating the need for additional ethical approval. The flowchart of the data extraction is shown in Fig. 1.

### Instrumental Variable (IV) selection

Multiple Single Nucleotide Polymorphisms (SNPs) representing genetic variations were chosen as Instrumental Variables (IVs) for bidirectional two-sample MR analysis. Effective IVs must satisfy three assumptions: (1) Strong association with exposure; (2) Influence on the outcome exclusively through the exposure; (3) No association with confounders of the outcome (Davies et al. 2018). Additionally, we adopted the following criteria to filter IVs, reducing potential interferences: (1) Significance threshold associated with IV: *p* < 5 × 10^−6^; (2) r^2^ < 0.001; (3) Physical distance between genes > 10,000 kb; (4) Exclusion of palindromic SNPs with intermediate allele frequencies; (5) Inclusion of IVs with an F-statistic greater than 10 to ensure sufficient correlation with exposure and to prevent biases due to weak IVs, where the F-statistic is calculated as F = R^2^(*N* − 2)/(1 - R^2^) (Burgess et al. 2011); (6) Screening for confounder-related IVs through genome-wide association studies.

### MR analysis

In MR analysis, the primary method used was the Inverse Variance Weighted (IVW) approach, complemented by MR Egger, Weighted Median, Simple Mode, and Weighted Mode algorithms to investigate the bidirectional causal relationships between MS and primary SS. The effect size was assessed using Odds Ratios (OR) and 95% Confidence Intervals (CI). The IVW method utilized the precision of each independent estimator as weights to calculate a combined estimate of all outcomes (Bowden et al. 2017).

### Sensitivity analysis

MR Pleiotropy RESidual Sum and Outlier (MR-PRESSO) method was employed to detect and adjust for pleiotropy arising from instrumental variable heterogeneity or outliers. Upon identifying outliers, MR-PRESSO corrected the causal estimates by excluding these outliers (Verbanck et al. 2018). Moreover, Cochrane’s Q statistic was used to assess heterogeneity among IVs; significant heterogeneity was considered present at *p* < 0.05, and a random-effects model was employed for effect estimation (Bowden et al. 2018; Kulinskaya and Dollinger 2015). For the assessment of horizontal pleiotropy, MR Egger regression was used, with the intercept term and its significance level indicating the presence of pleiotropy. Finally, a leave-one-out analysis was conducted to evaluate the influence of individual SNPs on the observed associations.

### Statistical analysis

Bonferroni correction was applied to interpret the multiple comparisons in the bidirectional tests (Sedgwick 2014). Post-Bonferroni correction, results with a p-value of < 0.025 were considered statistically significant. All statistical analyses were conducted using R software (Version 4.3.0) (Hemani et al. 2018) along with the TwoSampleMR (Version 0.5.7) and MR-PRESSO (Version 1.0) packages (Verbanck et al. 2018).

## Results

**Fig. 2:**
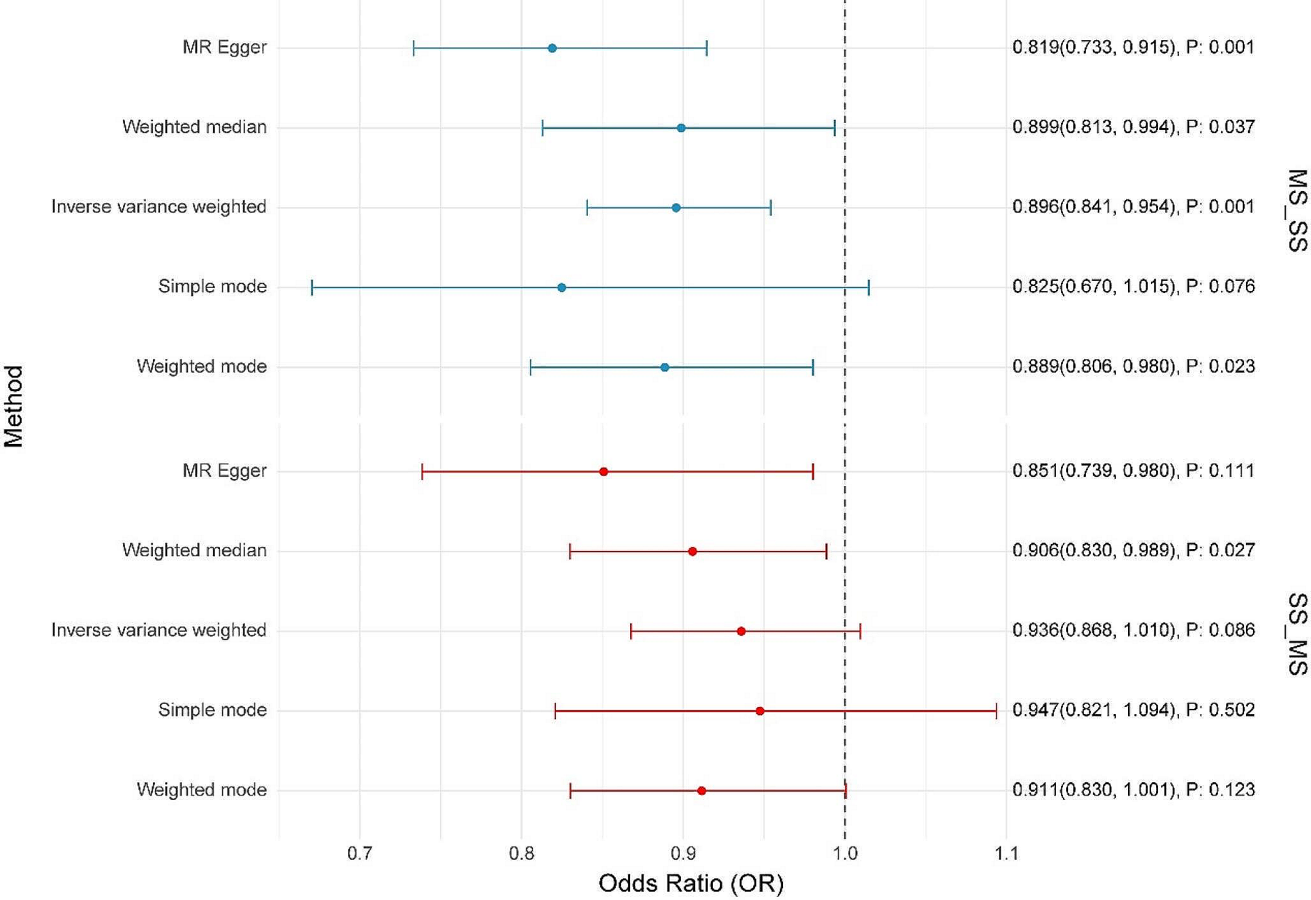
The results of a MR analysis comparing the causal effects of genetic predisposition to MS on the risk of primary SS and vice versa. The analysis was conducted using several statistical methods. Each method’s point estimate of the OR is represented. The values on the left suggest a protective effect and to the right indicate a risk factor. The plot suggests a potential protective effect of genetic predisposition to MS on the risk of primary SS and a non-significant effect of genetic predisposition to primary SS on the risk of MS.

**Fig. 3:**
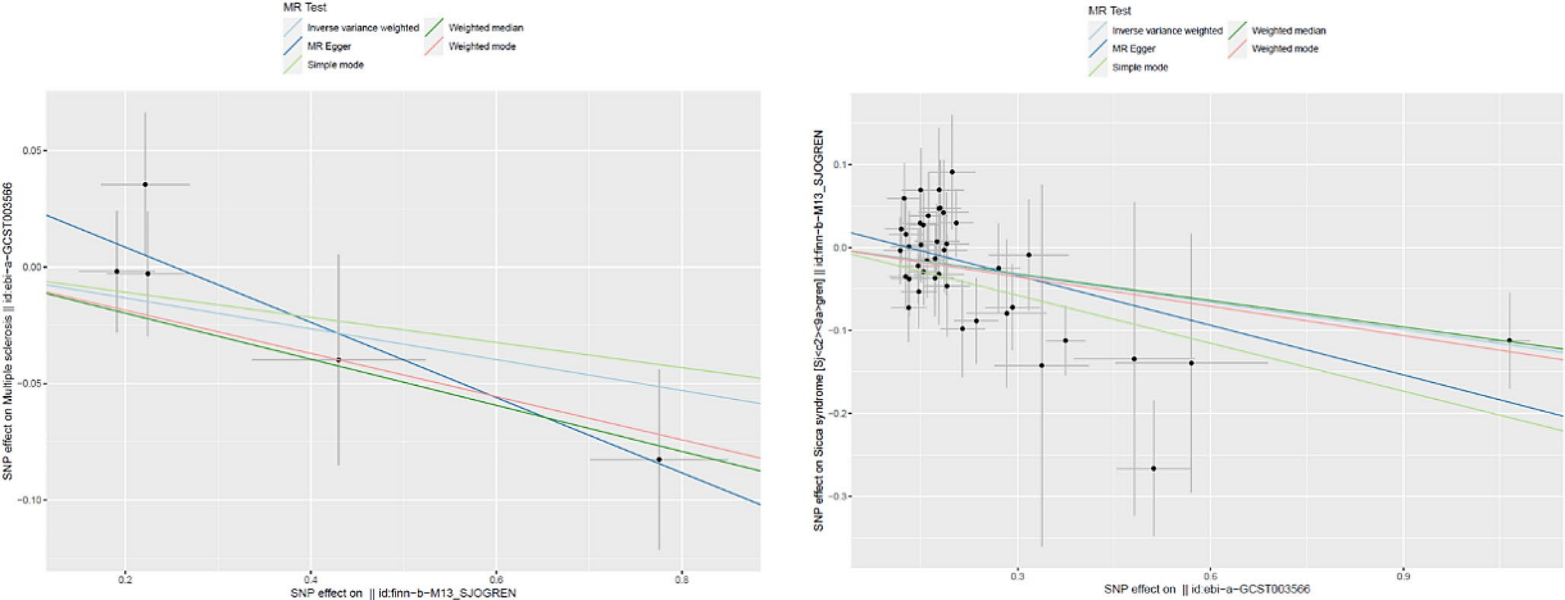
SNP effects on MS or primary SS using multiple methods. Points denote individual SNPs, while lines reflect different MR methods (MR-Egger, Weighted median, IVW, Simple mode). The slope indicates the causal effect magnitude, and the line positions reflect pleiotropy and estimate precision

In our study, after excluding SNPs with pleiotropic effects, we identified 42 and 5 SNPs as robust instrumental variables for primary SS and MS, respectively (Table S1, S2). Utilizing these instrumental variables, we applied multiple analytical methods to evaluate the potential causal effect of MS on primary SS. The IVW method revealed a significant protective effect of MS on primary SS, with an OR of 0.896 (95% CI 0.841–0.954; *P* = 0.001) (Figs. 2 and 3). The MR Egger method produced an OR of 0.819 (95% CI 0.733–0.915; *P* = 0.001) (Figs. 2 and 3), indicating that the protective effect of MS on primary SS remained significant after adjusting for multidirectional pleiotropy. Heterogeneity was not significant (Cochran’s Q-value = 40.714, *P* = 0.483), and no horizontal pleiotropy was detected (*P* = 0.060) (Table S3). Additionally, the weighted median approach showed an OR of 0.899 (95% CI 0.813–0.994; *P* = 0.037) (Figs. 2 and 3) for the simple model and 0.825 (95% CI 0.670–1.015; *P* = 0.076) (Figs. 2 and 3) for the weighted model, supporting the protective effect of MS against primary SS.

When assessing the potential causal effect of primary SS on MS, the IVW method indicated an OR of 0.936 (95% CI 0.868–1.010; *P* = 0.086) (Figs. 2 and 3), with no significant heterogeneity (Cochran’s Q-value = 3.771, *P* = 0.438) or evidence of horizontal pleiotropy (*P* = 0.216) (Table S3). Additional methods, including MR Egger and weighted median, yielded ORs of 0.851 (95% CI 0.739–0.980; *P* = 0.111) and 0.906 (95% CI 0.830–0.989; *P* = 0.027) (Figs. 2 and 3), respectively, further supporting our findings.

**Fig. 4:**
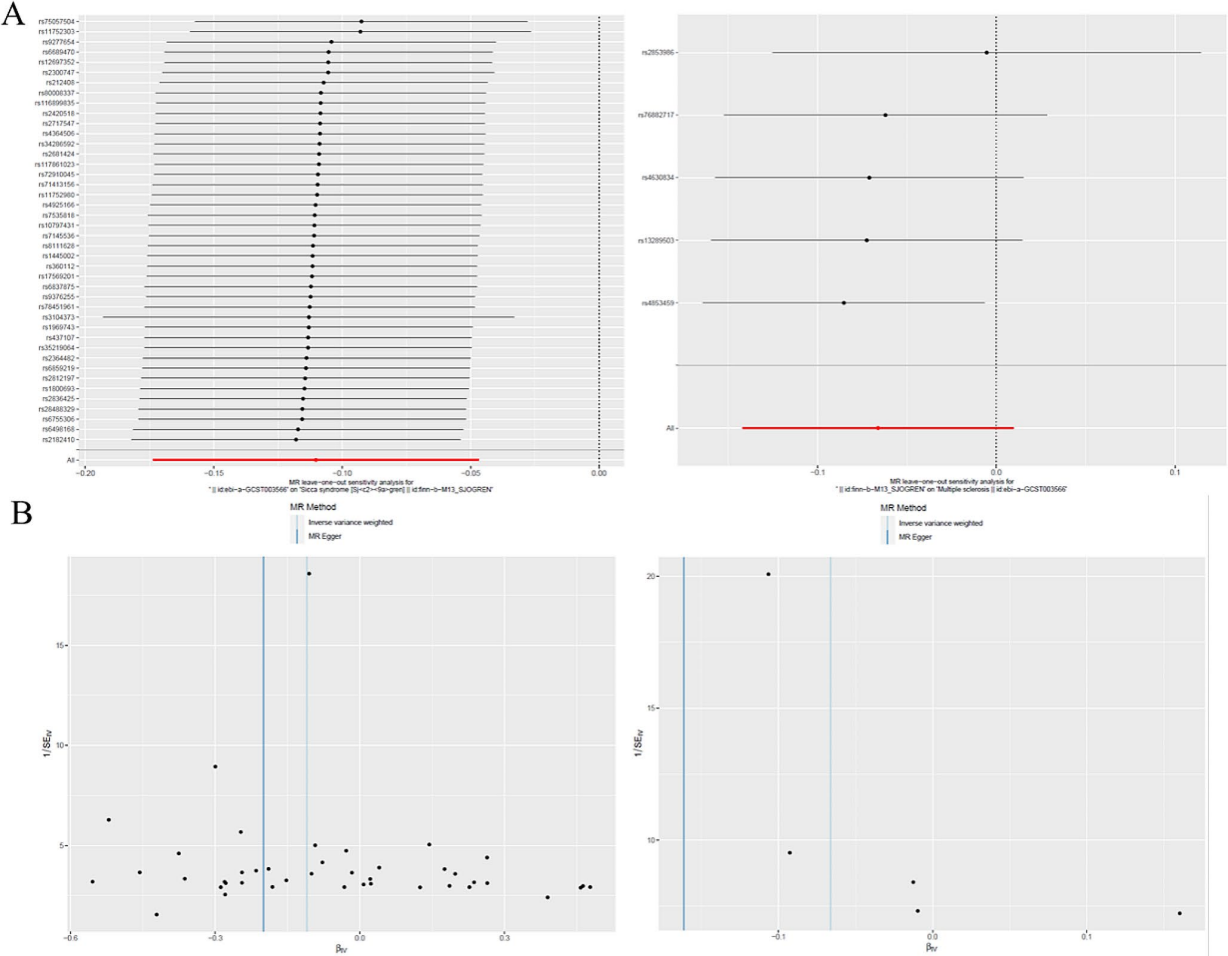
**A** leave-one-out (LOO) sensitivity analyses, where each point represents the recalculated effect size with the omission of that specific data point, highlighting the influence of individual SNPs on the overall effect estimate. **B** funnel plots, with the effect sizes on the horizontal axis and standard error on the vertical axis, used for detecting publication bias and heterogeneity, where symmetry suggests no bias

The MR-PRESSO global test indicated that the causal estimates of MS on primary SS and vice versa were not significantly influenced by any single SNP (Table S3). The leave-one-out analysis demonstrated that all instrumental variables influenced the outcomes in the same direction, aligning to the left of the null value (Fig. 4A). Furthermore, the funnel plot showed an even distribution of instrumental variables on both sides of the null value (Fig. 4B). These combined analyses suggest that MS may lower the risk of developing primary SS, indicating a potential protective effect against primary SS.

## Discussion

In this investigation, we assessed the potential protective effect of MS on primary SS using a MR approach, selecting 42 and 5 SNPs as instrumental variables for MS and primary SS, respectively. These SNPs were carefully chosen to minimize cross-sectional pleiotropy, adhering to the MR principle that genetic variants should affect the outcome solely through their impact on the exposure (Ebrahim and Davey Smith 2008). This methodological approach is in line with previous robust studies that have successfully uncovered causal relationships between various diseases and factors, such as the interplay between primary SS and gut flora (Cao et al. 2023), the linkages with cancers and other autoimmune diseases (Yeung et al. 2022), utilizing advanced statistical tools including IVW, MR Egger, and weighted median methods to ensure the integrity and accuracy of our analysis.

Our detailed analysis, employing a suite of MR methods, consistently demonstrated that MS significantly reduces the risk of primary SS. Specifically, the IVW method indicated a notable 10.4% decrease in risk, while the MR Egger method further emphasized this protective effect, showing an 18.1% reduction in risk. These results highlight the critical importance of selecting instrumental variables devoid of pleiotropic biases to make reliable causal inferences. Although the precise biological mechanisms underlying these observations are yet to be fully understood, the current literature points towards several key factors that could be instrumental, including the activity of the CXCL13/CXCR5 axis in autoimmune disorders (Pan et al. 2022) and the modulation of immune responses through mechanisms such as mitochondrial autophagy (Qiu et al. 2022), These insights suggest that MS may indirectly lower the risk of primary SS by influencing specific immune regulatory pathways, thus necessitating further experimental and clinical studies to validate these findings.

This study benefits from the use of pooled-level data and a variety of MR methodologies, enhancing the precision of our findings. Nonetheless, it is not without limitations. Despite rigorous attempts to adjust for pleiotropic biases through methods like MR-Egger regression and MR-PRESSO global tests, the potential for residual pleiotropic effects remains, which could limit the accuracy of our causal inferences. Additionally, our reliance on summary-level data means we are unable to conduct detailed subgroup analyses or assess inter-individual heterogeneity comprehensively. Moreover, while we have endeavored to select SNPs with minimal pleiotropy as instrumental variables, the complex nature of genetic variation may still impact the robustness of our conclusions. The applicability of our findings also warrants caution; the generalization of these insights calls for further empirical validation through biological studies. In conclusion, while this study offers pioneering insights into the protective role of MS against primary SS, its interpretation and application should be approached with prudence. Future research is essential to address the current study’s limitations and to delve deeper into the causal relationship and biological mechanisms linking MS and primary SS, potentially paving the way for novel therapeutic strategies.

## Conclusion

In this study, MS was found to have a potentially protective role in reducing the risk of primary SS through MR analyses, providing genetic evidence to support a possible negative causal association between the two diseases. This finding emphasizes the value of genetic approaches in unraveling potential interactions between diseases and provides new perspectives for future biomarker research and therapeutic strategy development.

## Data Availability

No datasets were generated or analysed during the current study.
